# IgG response to spike protein of SARS-CoV-2 in healthy individuals and potential of intravenous IgG as treatment for COVID-19

**DOI:** 10.1186/s12985-022-01921-z

**Published:** 2022-11-13

**Authors:** Gang Wang, Zebao He, Fengtian Wu, Zhengming Ge, Jiansheng Zhu, Zhi Chen

**Affiliations:** 1 State Key Laboratory for Diagnosis and Treatment of Infectious Diseases, National Clinical Research Center for Infectious Diseases, Collaborative Innovation Center for Diagnosis and Treatment of Infectious Diseases, The First Affiliated Hospital, College of Medicine, Zhejiang University, 79 Qingchun Road, Hangzhou, 310003 Zhejiang Province China; 2 Department of Infectious Diseases, Taizhou Hospital of Zhejiang Province Affiliated to Wenzhou Medical University, No.150 Ximen Road of Linhai City, Taizhou, 317000 China; 3 Department of Infectious Diseases, Enze Hospital, Taizhou Enze Medical Center (Group), Taizhou, China

**Keywords:** COVID-19, SARS-CoV-2, Common coronavirus, IVIg, Cross seropostivity

## Abstract

**Background:**

The severe acute respiratory syndrome coronavirus-2 (SARS-CoV-2) is the cause of coronavirus disease 2019 (COVID-19), which is currently a worldwide pandemic. There are limited available treatments for severe COVID-19 patients. However, some evidence suggests that intravenous immunoglobulin (IVIg) provides clinical benefits for these patients.

**Methods:**

We administered IVIg to 23 severe COVID-19 patients, and all of them survived. Four related coronaviruses can cause the common cold. We speculated that cross-reactivity of SARS-CoV-2 and other common coronaviruses might partially explain the clinical efficacy of IVIg therapy. Thus, we performed multiple alignment analysis of the spike (S), membrane (M), and nucleotide (N) proteins from SARS-CoV-2 and the common coronaviruses to identify conserved regions. Next, we synthesized 25 peptides that were conserved regions and tested their IVIg seropositivity.

**Results:**

The results indicated four peptides had significant or nearly significant seropositivity, and all of them were associated with the S and M proteins. Examination of the immune responses of healthy volunteers to each synthetic peptide indicated high seropositivity to the two peptides from S protein. Blood samples from healthy individuals may have pre-existing anti-SARS-CoV-2 IgGs, and IVIg is a potentially effective therapy for severe COVID-19.

**Conclusion:**

In conclusion, blood samples from many healthy individuals have pre-existing anti-SARS-CoV-2 IgGs, and IVIg may be an effective therapy for severe COVID-19.

**Supplementary Information:**

The online version contains supplementary material available at 10.1186/s12985-022-01921-z.

## Introduction

The novel coronavirus disease 2019 (COVID-19) is caused by the severe acute respiratory syndrome coronavirus-2 (SARS-CoV-2), which is in the family *Coronaviridae*, and subfamily *Coronavirinae*. SARS-CoV-2 can infect humans and other vertebrates [1], and transmission among humans is mainly via respiratory droplets following direct human-to-human contact, although transmission can occur by other routes [2]. Because the virus is highly contagious, COVID-19 has spread throughout China and Europe, and then worldwide, resulting in more than 90 million people with the disease and more than 2 million deaths worldwide by the end of 2020 (data from coronavirus resource center in Johns Hopkins University)[3]. In March 2020, the WHO declared COVID-19 a pandemic and worldwide public health emergency.

SARS-CoV-2 and six other coronaviruses (229E, NL63, HKU1, OC43, SARS, and MERS) can cause diseases in humans [4, 5]. The 2003 SARS epidemic and the 2012 MERS epidemic were caused by two different highly pathogenic coronaviruses and led to thousands of deaths [6]. Unlike SARS and MERS, the four other coronaviruses (229E, NL63, HKU1, and OC43) have been circulating in humans for centuries, and their infections of the respiratory tract only lead to mild symptoms, such as the common cold. Coronavirus infections trigger the host to produce immunoglobulins (Igs) [6]. Hence we speculate that many human populations have pre-existing Ig responses to infection by common coronaviruses.

Several vaccines are now available for preventing the COVID-19 pandemic, but only limited treatments are available. There are increasing efforts to develop novel antiviral agents against SARS-CoV-2, and one possible approach is the administration of intravenous Ig (IVIg) [2, 7]. IVIg consists of blood proteins derived from the plasma of healthy donors, and this therapy is commonly used to treat numerous conditions [6]. In particular IVIg therapy can improve the clinical outcomes of patients with numerous inflammation-associated diseases, such as rheumatic diseases, organ-specific autoimmune diseases, atopic diseases, and neurological disorders [6, 7]. This therapy can also improve the clinical outcomes of patients with SARS and MERS [8–10].

According to the WHO, about 80% of confirmed COVID-19 patients develop mild-to-moderate disease, 13.8% develop severe disease (characterized by dyspnea), and 6.1% develop critical symptoms, such as respiratory distress syndrome concomitant with cytokine release syndrome, septic shock, and multiple organ dysfunction or failure (https://www.who.int). A previous study of COVID-19 patients reported IVIg therapy had anti-inflammatory effects due to its scavenging of complement [11], blocking receptors of immune cells [11], and direct anti-viral effects [7, 12]. However, the mechanism of IVIg therapy is still uncertain, and its effectiveness in treating patients who are critically ill with severe COVID-19 is uncertain.

We hypothesized that there should be some cross-reactive antibody responses to epitopes that are shared by the four common coronaviruses that are currently in circulation and SARS-CoV-2. Thus, we examined this hypothesis by identification pre-existing Igs against conserved peptide sequences from the Spike (S) and membrane (M) proteins of SARS-CoV-2 in a healthy population.

## Materials & methods

### Patients and healthy volunteers

From Jan 11 to Feb 28, 2020, 23 patients with severe COVID-19 (confirmed by RT-PCR and chest computed tomography [CT]) were enrolled, all of whom were in the intensive care unit (ICU) at Taizhou Central Hospital, Zhejiang Province. Disease severity was determined by the 7th edition of the “diagnosis and treatment guidelines of coronavirus 2019 in China” [13]. The data of these patients were collected retrospectively and included demographic characteristics, clinical records, laboratory data, chest CT results, and information collected at ICU admission.

An additional 197 healthy volunteers were recruited from the First Affiliated Hospital of Zhejiang University School of Medicine at the same time. These healthy volunteers were more than 20 years old, in good health, had no history of blood transfusion or monoclonal antibody therapy, and were free of infection by SARS-CoV-2 (Nucleic acid-based PCR) and the virus specific Igs (IgG/E/M). These healthy adults were medically healthy based on clinical history, physical examination, negative routine blood tests, routine urine tests, and thoracic X-ray results.

This study was approved by the Ethics Committee of Taizhou Central Hospital and the First Affiliated Hospital, Zhejiang University School of Medicine.

### Selection of antigenic peptides

Polyprotein sequences of the structural proteins of coronaviruses responsible for the common cold (229E, NL63, OC43, and HKU1) and SARS-CoV-2 were obtained from the NCBI database (Additional file 1: Table S1). These protein sequences of the coronaviruses were performed with Vector NTI (version 11.5) (Thermo, USA) to find the evolutionally conserved amino acid residues. Based on this, some peptide sequences were included, which were conserved sequences or closed to conserved sequences. All the peptides were conjugated with biotin at the C-terminal and synthesized by a company (Sangon Biotech (Shanghai) Co., Ltd).

### Enzyme-linked immunosorbent assay (ELISA)

IVIg was purchased from Sichuan Yuanda Shuyang Pharmaceutical Company (> 98% IgG, Batch No.: 201908143B, 100 mg/mL) and this batch was produced before the break of COVID-19 (08/2019). ELISA was performed using streptavidin-coated 96-well ELISA plates (Cat: 45,360, R&D Systems) according to the manufacturer's instructions [14]. Briefly, 100 μL of biotinylated peptides (1 μg/mL) was added to each well, covered with adhesive strip, and incubated overnight at room temperature. After three washes (350 μL/well of wash buffer), 100 μL of 1/10 dilution of IVIg or serum of healthy donors was added. After 2 h at room temperature, three aspirations/washes were performed. Then HRP-coupled anti-Fc of human IgG (1:10,000 in PBS) was added to each well. After 1 h at room temperature, chromogen solutions A and B were added. Finally, 3 to 5 min later, a stop solution was added and the absorbance was measured at 405 nm using a microplate reader. Positive and negative antigen wells were used as controls. There were 6 replicates for each sample and 10 replicates for each control.

### Statistical analysis

All statistical analyses were conducted using SPSS version 23 (IBM). Student’s *t*-test or the chi-square test was used for comparisons of different groups, as appropriate. A P-value below 0.05 was considered significant.

## Results

### IVIg improved the clinical outcomes of severe COVID-19 patients

**Fig. 1:**
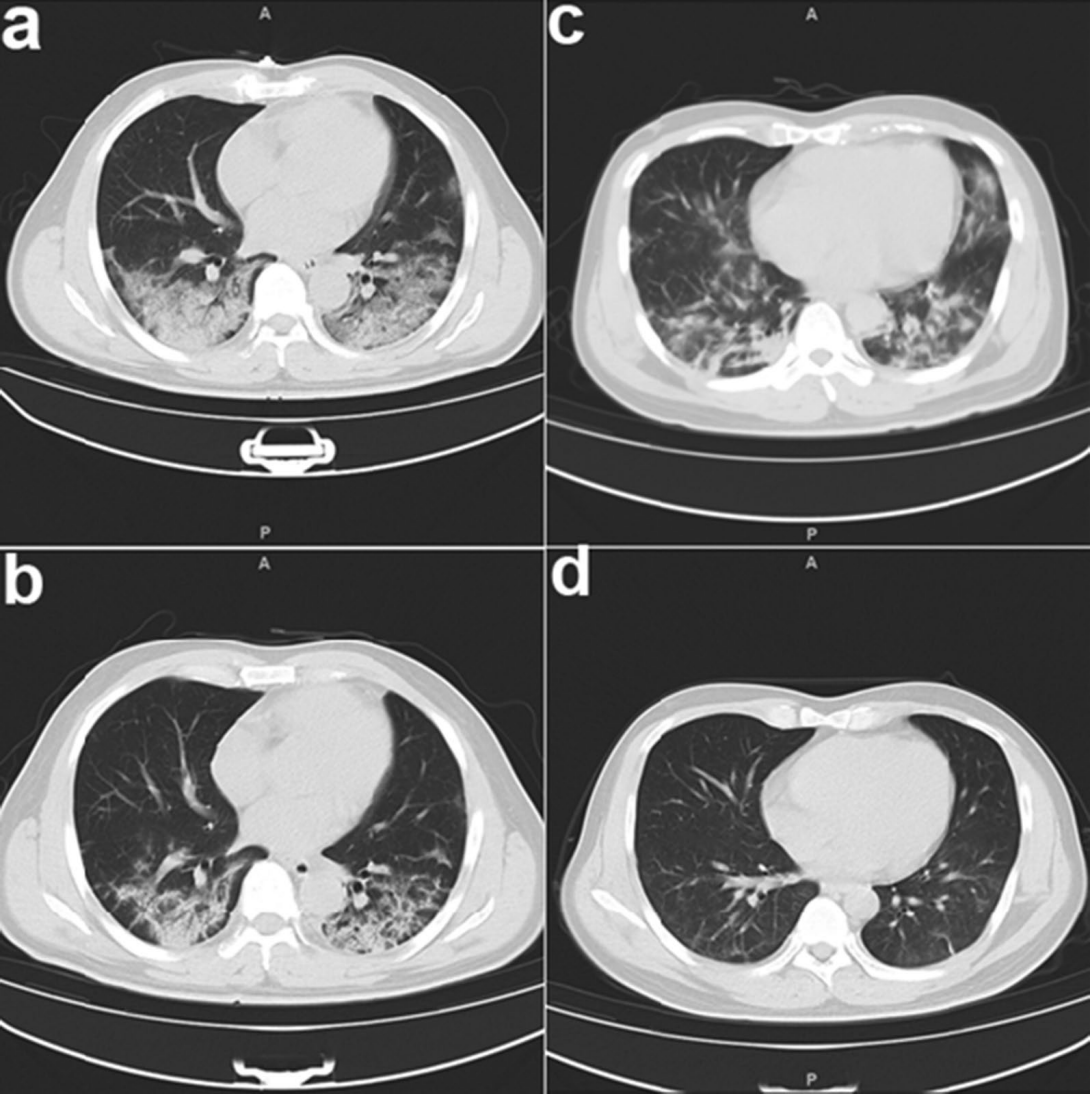
CT scans of the lungs of two representative patients with severe COVID-19 before treatment (**A** and **C**) and after treatment (**B** and **D**). Note the reduced lesion size and inflammation after treatment

Previous research demonstrated that IVIg therapy reduced the symptoms and improved the clinical outcomes of patients with severe COVID-19 [9, 10, 15, 16]. We examined 23 patients with critical/severe COVID-19 who were in the ICU of our hospital (Table 1). All patients received high dose IVIg (0.4 g/kg body weight) concomitant with a glucocorticoid (40 mg/day) for 3 to 5 days. Anti-viral treatments (interferon, lopinavir-ritonavir, and arbidol) were also administered. After these intensive treatments, all patients experienced symptom relief, including increased blood oxygen saturation, decreased body temperature, and normalization of lymphocyte count. Moreover, CT analysis showed significantly reduced lung lesions and inflammation after 5 days (Fig. 1). A previous study reported the mortality rate was about 28–48% in patients with severe COVID-19 [9, 17]. However, there were no deaths among our 23 patients. This suggests that treatment with IVIg and other drugs and antiviral agents may improve the clinical outcome and the reduced mortality of patients with severe COVID-19.

**Table 1:** Characteristics of patients with severe COVID-19 (n = 23)

Characteristics	Total number
Age(range)	53.52 ± 13.19 (30–75)
Gender	
Male	14(60.87%)
Female	9(39.13%)
Comorbidity	
Hypertension	3(13.04%)
Coronary heart disease	1(4.34%)
Diabetes	3(13.04%)
Carcinoma	2(8.70%)
Symptoms	
Fever(temperature ≥ 37.3 °C)	19(82.61%)
Cough	20(86.96%)
Haemoptysis	1(4.34%)
Sputum	15(65.22%)
Dyspnoea	12(52.17%)
Nausea or vomiting	3(13.04%)
Diarrhea	4(17.39%)
Myalgia	9(39.13%)
PSI Score	
III (General)	1(4.34%)
IV (Severe)	21(91.30%)
V (Critical)	1(4.34%)
Laboratory main findings	
White blood cell count, × 10^9^ per L	
< 4	3(13.04%)
4–10	19(82.61%)
> 10	1(4.34%)
Lymphocyte count, × 10^9^ per L	
< 0.8	12(52.17%)
Haemoglobin, g/L	138.43 ± 17.85
Platelet, × 10^9^ per L	197.30 ± 65.93

*PSI* pneumonia severity index

### Antibodies in the IVIg recognize peptides from the M and S proteins of *SARS-CoV-2*

We hypothesized that antibodies in the IVIg against 4 common coronaviruses (229E, OC43, HKU1, and NL63) were cross-reactive to similar epitopes on SARS-CoV-2. Thus, we aligned the amino acid sequences of the S, M, and E proteins of SARS-CoV-2 with sequences in 229E, OC43, HKU1, and NL63. The results showed similarities of the E, M, and S proteins among these coronaviruses. In particular, there were two regions of conserved sequences that were similar to the E protein (Additional file 1: Fig. S1) and many conserved sequences were similar to the M and S proteins (Additional file 1: Fig. S2 and Additional file 1: Fig. S3). Most of the conserved residues of the S protein were in the C-terminal region (Additional file 1: Fig. S3). Overall, we selected 25 conserved amino acid sequences for further analysis (Additional file 1: Table S1).

**Fig. 2:**
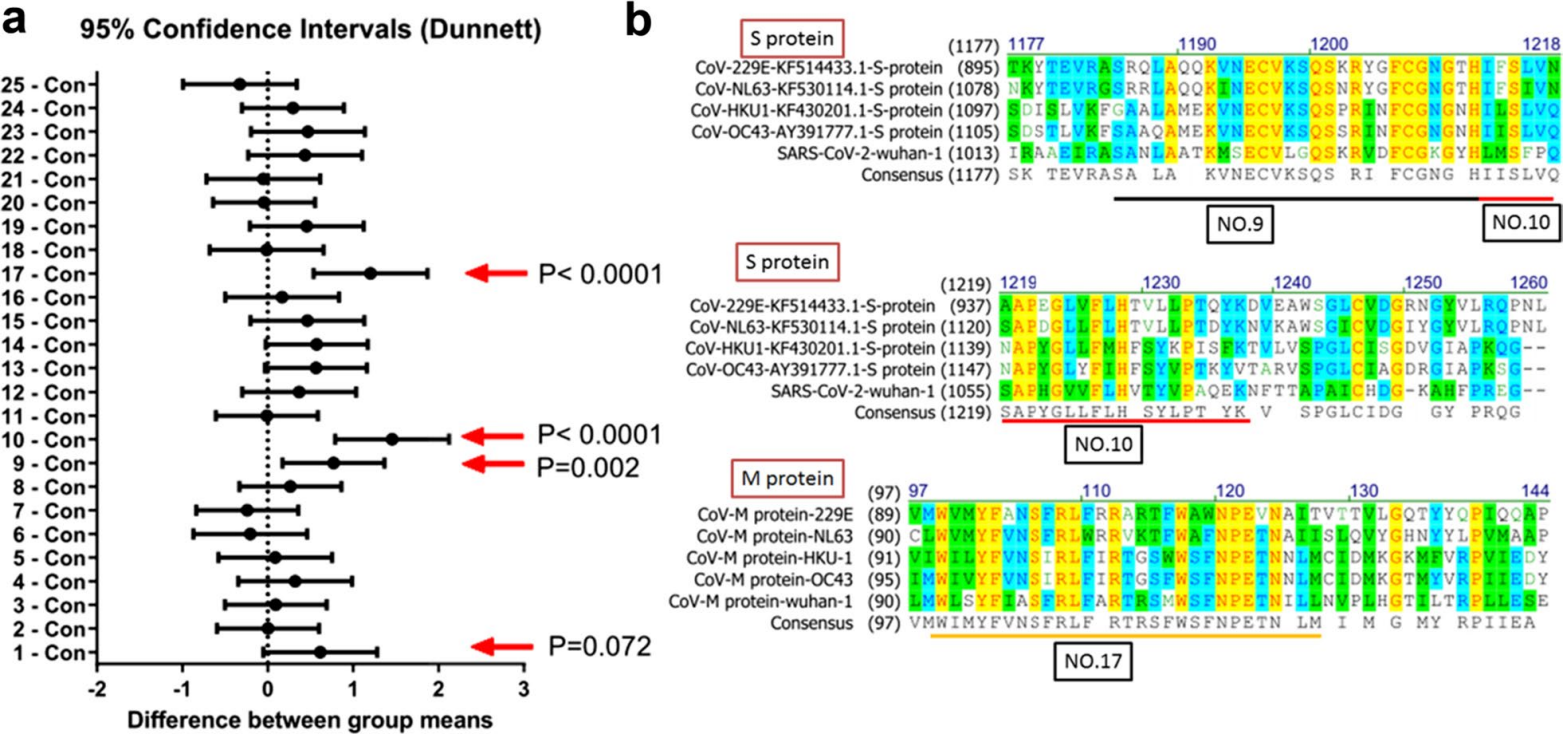
Responses of IVIg to 25 different synthetic peptides (**A**) and sequence comparisons of synthetic peptides with the S and M proteins of SARS-CoV-2 (**B**). Note the significant responses to peptides No. 9, No. 10, and No. 17, and the nearly significant response to peptide No. 1

The 25 synthesized peptides were conjugated with biotin and added to a streptavidin-coated ELISA plate. After blocking with serum, the plates were incubated with IVIg and stained with a HRP-secondary antibody. The results indicated that peptides No. 9 (P = 0.002), No. 10 (P < 0.0001), and No. 17 (P < 0.0001) had significantly greater binding than control wells (Fig. 2A). Additionally, peptide No. 1 had nearly significant binding (P = 0.072). Further analysis showed that peptides No. 1, No. 9, and No. 10 peptides were similar to regions in the S protein (Aa91-120, Aa1021-1048, and Aa1049-1074, respectively), and No. 17 was similar to a region in the M protein (Fig. 2B). Taken together, these results suggest that IVIg reacts to SARS-CoV-2 antigens by recognizing conserved epitopes.

### Synthetic peptides elicit responses in plasma from healthy individuals

**Fig. 3:**
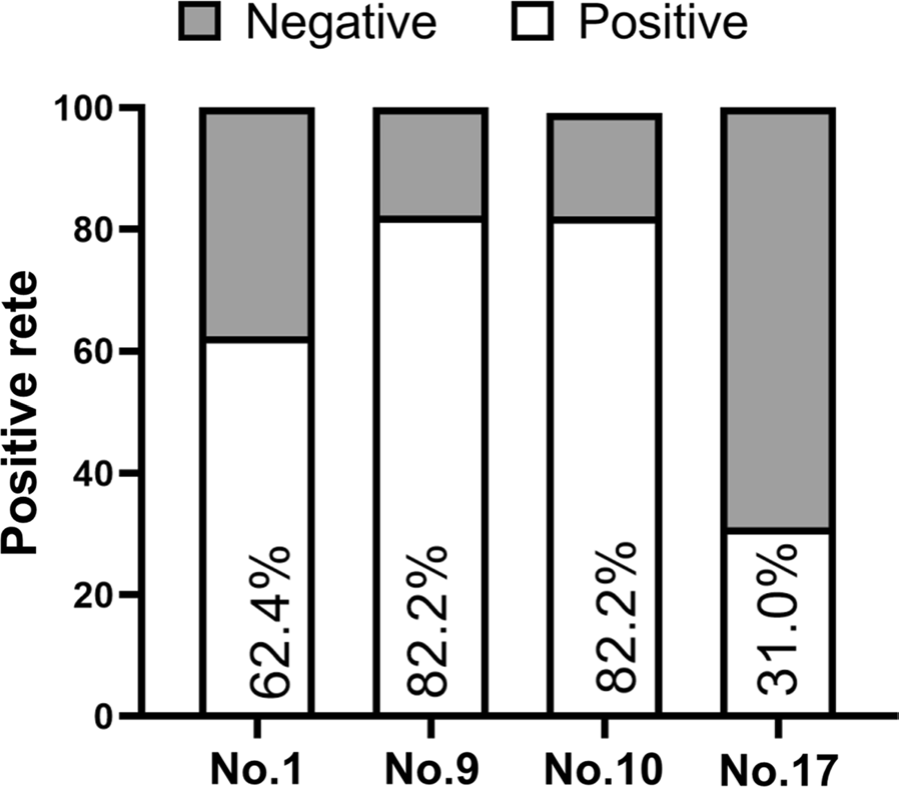
Seropositivity rates of healthy volunteers (n = 197) to four different synthetic peptides. Note the high seropositivity rates for peptides No. 9 and No. 10, which correspond to the S protein of SARS-CoV-2

We examined whether healthy individuals had antibodies that responded to the 4 peptides identified above. Thus we collected 197 plasma samples from healthy subjects for ELISA testing. The results indicated that 62.4% were seropositive to peptide No. 1, 82.2% were seropositive to peptide No. 9, 82.2% were seropositive to peptide No. 10, and 31.0% were seropositive to peptide No. 17 (Fig. 3). These results suggested the positive rates were higher than that of SARS-CoV-2-specific T cell [18].

Previous research showed that males had increased the risk for severe COVID-19 [19, 20]. We therefore compared the serological response to peptides No. 9 and No. 10 in males and females. The chi square test indicated that females had a significantly greater seropositivity to peptide No. 10 (82.18% vs. 61.46%, P = 0.015) (Table 2).

**Table 2:** Effect of age and gender of healthy subjects (n = 197) on seropositivity to peptides No. 9 and No. 10

	Healthy individuals (197)			
Peptide	No.9		No.10	
Groups	< = 50y	> 50y	Female	Male
Positive	117(79.05%)	45(91.84%)	83(82.18%)	59(61.46%)
Negative	31(20.95%)	4(8.16%)	18(17.82%)	37(38.54%)

Several other reports indicated that older COVID-19 patients had an increased risk for mortality [21, 22]. Thus, we also compared the seropositivity rates to peptide No. 9 in different age groups (Table 2). The results showed a higher rate of seropositivity in individuals elder than 50 years (91.84% vs. 79.05%, P = 0.042), but there was no significant difference for the other peptides (data not shown).

## Discussion

There have been pulses of increasing of SARS-CoV-2 infected cases in many countries, including China where the COVID-19 pandemic was controlled. Although the pandemic has lasted more than 1 year and there are several effective vaccines, there are limited effective therapeutics. Different therapeutic approaches have been examined by clinicians in different regions. One promising treatment against COVID-19 is IVIg, whether alone or in combination with antivirals or corticosteroids.

In the present work, we treated 23 ICU patients who had severe COVID-19 using IVIg in combination with other antiviral agents. All 23 of our patients survived, suggesting that IVIg concomitant with other therapeutics may be an effective treatment for patients with severe COVID-19. However, there is a limitation to using IVIg to treat all the COVID-19 patients. Thus, the effectiveness of IVIg for treating COVID-19 patients is still needed further clinical studies with control groups. We therefore examined the possible mechanism of this effect. We first identified three peptides from the SARS-CoV-2 S protein (No. 1, No. 9 and No. 10) and one from the SARS-CoV-2 M protein (No. 17) that elicited serological responses in the plasma of healthy individuals. These results suggested that antibody responses to epitopes of SARS-CoV-2 are pre-existing in individuals who were not infected with SARS-CoV-2. These four peptides are in evolutionarily conserved regions of proteins in SARS-CoV-2 and in four related coronaviruses that are responsible for the common cold. These four common coronaviruses are widespread and have been circulating for many years in human populations, where they account for about 30% of all cases of the common cold [23]. Thus, most people who have experienced the common cold may have immune memory for coronaviruses, especially those infected with the widespread 229E and OC43 coronaviruses [18, 24, 25]. These results explain the reason for the pre-existence of antibodies against SARS-CoV-2 in unexposed human populations.

Several studies reported that high dose IVIg can relieve the symptoms of patients with severe COVID-19, increase the oxygen saturation and lymphocyte count, and reduce inflammation [9, 10, 15, 16]. More specifically, Shao et al. conducted a multicenter retrospective cohort study of patients with COVID-19 and found reduced mortality in the 174 patients who received IVIg relative to the 151 controls who did not [16]. Another study found that COVID-19 patients who received IVIg had reduced mortality relative to controls (20% vs. 48.5%) [9]. However, there still several new studies reporting that IVIg didn’t increase the benefits of severe COVID-19 patients or moderate-to-severe acute respiratory distress syndrome (ARDS) patients [26, 27]. Hence, whether the IVIg is effective for treating COVID-19 patients or not needs more clinical data. Next, we synthesized 25 peptides in the conserved regions of structural proteins of SARS-CoV-2 and confirmed that 4 of them elicited significant immune responses in the plasma of healthy subjects. This indicates the presence of antibodies that respond to SARS-CoV-2 in healthy individuals. Three of the peptides correspond to the S2 subunit of the S protein. Peptide No. 1 peptide is close to the cleavage sites of S1/S2 and S2’ and peptides No. 9 and No. 10 are between the two heptad repeats (HR1 and HR2) and are important for the conformation of the trimer of the S protein [28]. However, peptide No. 17 corresponds to the M protein, the amino acid residues of which ranges from the transmembrane region to the intra-membrane region.

In contrast to our results, Schwaiger et al. reported that antibodies against SARS-CoV-2 were not present in IVIg prepared from healthy populations before the COVID-19 pandemic [29]. These authors examined 54 lots of IVIg from the U.S. and Europe and concluded there were no functional neutralizing antibodies based on titration of neutralization using Vero cells [29]. However, their data had two issues that should be considered. First, the titer of the neutralizating antibodies may have been below their detection limits; and second, although antibody responses to epitopes of virus, the residues of the epitopes are not involved in the process of virus binding and entry into cells, perhaps meaning the antibodies could not interrupt the entry of the virus. Additionally, they found a high titer of neutralizing antibodies to 229E, a common cold coronavirus. These results therefore do not exclude our hypothesis that numerous cross-reactive antibodies to SARS-CoV-2 are present in healthy individuals due to their previous exposure to coronaviruses that cause the common cold.

Our results indicated the plasma of healthy females had higher seropositivity than males to peptide No. 10 (an S protein epitope). Previous research reported that the incidence of COVID-19 was lower in females than males [21, 30–33]. Moreover, we found that healthy older individuals had higher seropositivity than younger individuals to peptide No. 9 peptide. However, advanced age is considered a major risk factor for mortality in patients with severe COVID-19 [21]. The mechanisms underlying the relationships of pre-existing seropositivity in healthy individuals with gender and age require further exploration. An intriguing possibility is that cross-active antibodies may function as blocking agents. Additionally, the direct-anti-virial effects of IVIg could be examined in future, which is one of the limitations of the present work.

## Conclusion

The results of the present study and other published data confirmed the clinical benefit of IVIg. We also found that the IVIg had an active antibody response to peptides from the S protein of SARS-CoV and that healthy individuals (without SARS-CoV-2 infections) had immune responses to epitopes of the S protein of SARS-CoV-2. However, the peptides need to be specific for the epitopes recognized by IgG in humans. Future studies are needed to validate these results by examination of a large cohort of healthy individuals. These results could be precision medicine tools which can accelerate the understanding and treating of COVID-19.

## Supplementary Information

**Additional file1**. **Table S1**. The ID ofeach referenced coronavirus. **Fig. S1**. Envelopprotein alignment. **Fig. S2**. Membrane protein alignment. **Fig. S3** Spikprotein alignment 

## Data Availability

All data generated or analyzed during this study are included in this published article and its supplementary information files.

## Abbreviations

SARS: Severe acute respiratory syndrome coronavirus
MERS: Middle east respiratory syndrome
SARS-CoV-2: Severe acute respiratory syndrome coronavirus-2
COVID-19: Coronavirus disease 2019
IVIg: Intravenous immunoglobulin
S: Spike
M: Membrane
N: Nucleotide
CT: Computed tomography
ICU: Intensive care unit
ELISA: Enzyme-linked immunosorbent assay
