# Supplementary material for: IgG response to spike protein of SARS-CoV-2 in healthy individuals and potential of intravenous IgG as treatment for COVID-19
Source: Virol J. 2022 Nov 13;19:186. doi: 10.1186/s12985-022-01921-z (PMC9655819; doi:10.1186/s12985-022-01921-z)
Supplement: Supplementary file 1 — Additional file1. Table S1. The ID ofeach referenced coronavirus. Fig. S1. Envelopprotein alignment. Fig. S2. Membrane protein alignment. Fig. S3 Spikprotein alignment [file 12985_2022_1921_MOESM1_ESM.docx]

Supplementary files

S.Table 1 the ID of each referenced coronavirus

Fig.S1 Envelop protein alignment

Fig.S2 Membrane protein alignment

Fig.S3 Spike protein alignment

S.Table 1 the ID of each referenced coronavirus

| Coronavirus Name | NCBI Reference Sequence NO. |
| --- | --- |
| CoV-229E | KF514433.1 |
| CoV-NL63 | KF530114.1 |
| CoV-HKU1 | KF430201.1 |
| CoV-OC431 | AY391777.1 |
| SARS-CoV-2-wuhan-1 | NC_045512.2 |

Fig.S1 Envelop protein alignment


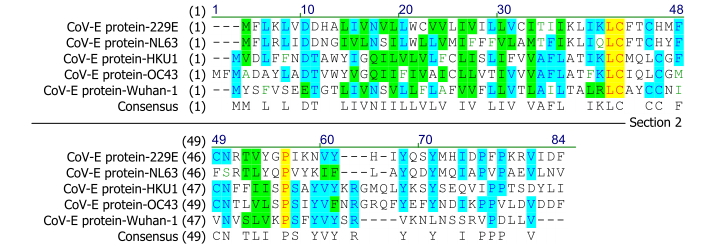


Yellow means completely conserved amino acids residues; Blue and green mean partially conserved among coronaviruses.

Fig.S2 Membrane protein alignment


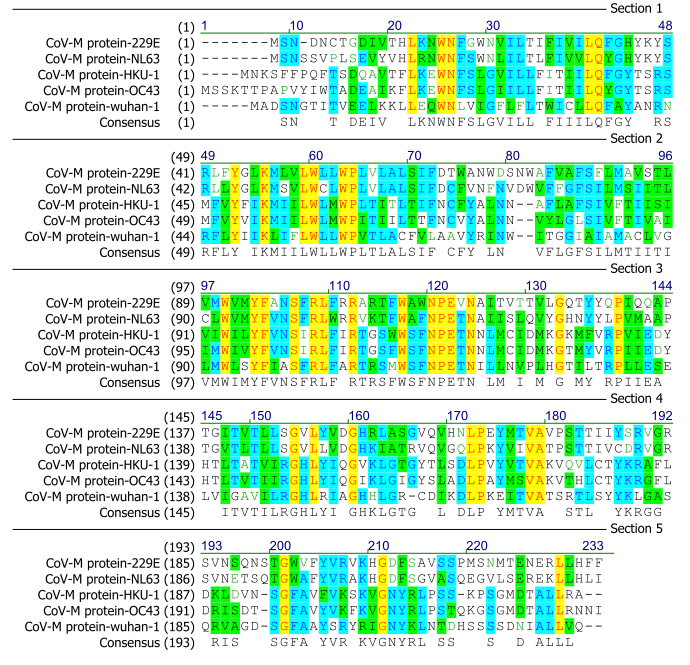


Yellow means completely conserved amino acids residues; Blue and green mean partially conserved among coronaviruses.

Fig.S3 Spike protein alignment


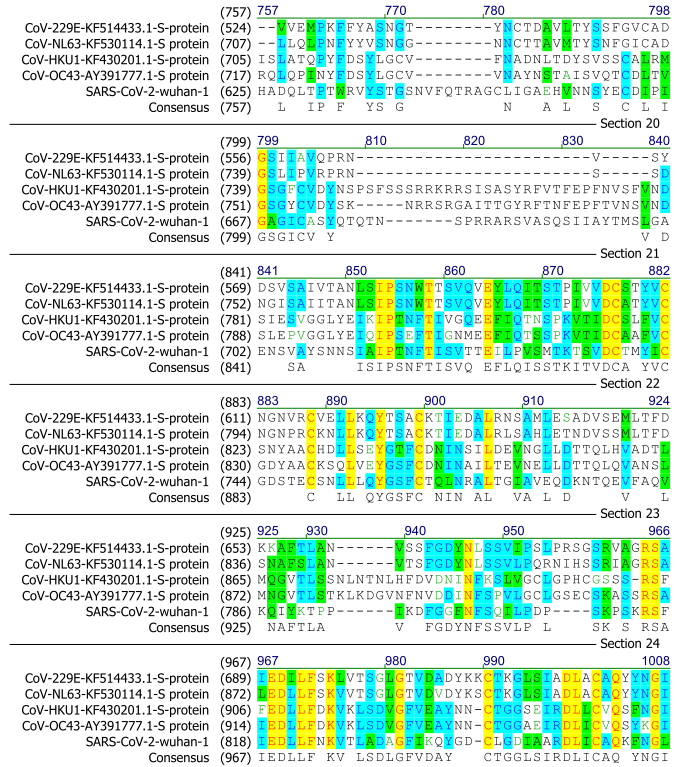


Yellow means completely conserved amino acids residues; Blue and green mean partially conserved among coronaviruses.

Continued


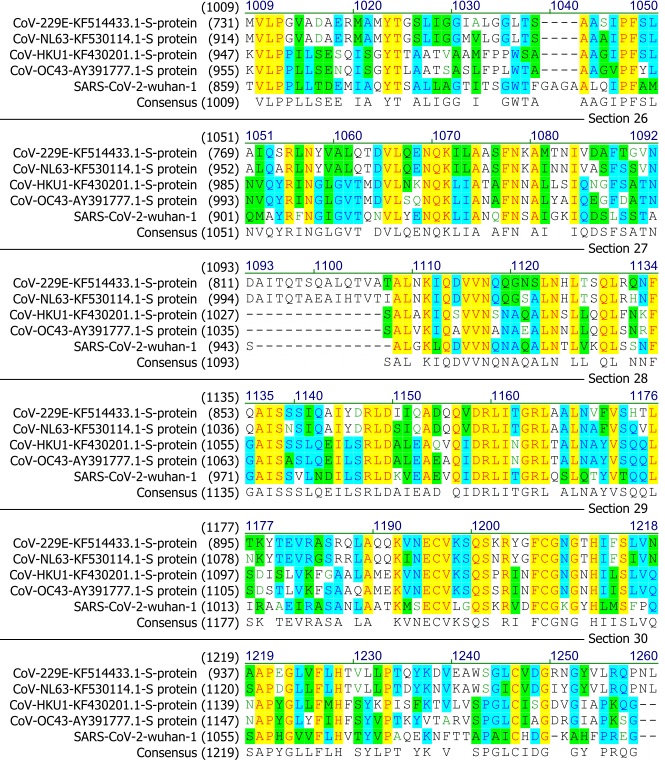


Continued


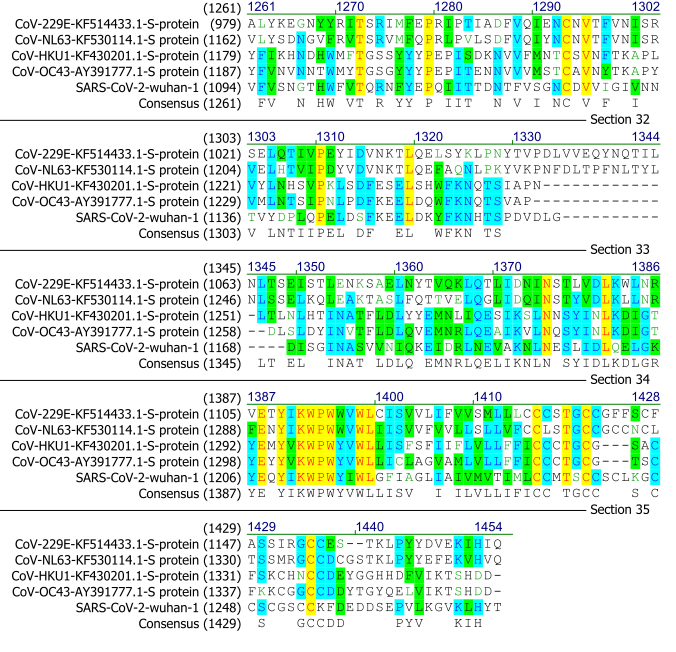


Table S1 Selected peptides from conserved sequence regions according to multiple alignment

| NO. | Proteins | Pepties |
| --- | --- | --- |
| 1 | S | TSVDCTMYICGDSTECSNLLLQYGSFCTQ |
| 2 |  | FGGFNFSQILPDPSKPSKRSFIEDLLFNK |
| 3 |  | IEDLLFNKVTLADAGFIKQYGDCLGDIAA |
| 4 |  | RDLICAQKFNGLTVLLPPLLTDEMIAQYT |
| 5 |  | GAALQIPEAMQMAYRFNGLGVTQNVLYEN |
| 6 |  | TAALGKLQDVVNQNAQALNTLVKQLSSNF |
| 7 |  | SNFGAISSVLNDILSRLDKVEAEVQIDRL |
| 8 |  | KVEAEVQIDRLITGRLQSLQTYVTQQLIR |
| 9 |  | SANLAATKMSECVLGQSSKRVDFCGKGYH |
| 10 |  | LMSFPQSAPHGVVFLHVTYVPAQEKN |
| 11 |  | APAICHDGKAHFPREGVFVSN |
| 12 |  | KNLNESLIDLQELGKYEQYIKWPWYIWLG |
| 13  14 |  | QNVLYENQKLIANQFNSAIGKIQDSLSST |
|  | M | RNRFLYIIKLIFLWLLWPVTLAC |
| 15 |  | LMWLSYFIASFRLFARTRSMWSFNPETNI |
| 16 |  | ILRGHLRIAGHHLGRCDIKDLPKEITVAT |
| 17 |  | MWLSYFIASFRLFARTRSMWSFNPETNILL |
| 18 |  | NRFLYIIKLIFLWLLWPVTLACFVL |
| 19 |  | DLPKEITVATSRTL |
| 20  21 |  | GAVILRGHLRIAGHHLG |
|  | E | PRWYFYYLGTGPEAGLPYG |
| 22 |  | ANKDGIIWVATEGALN |
| 23 |  | SKKPRQKRTA |
| 24 |  | QGTDYKHWPQIAQFAPSASAFFG |
| 25 |  | TKAYNVTQAFGR |

S, Spike protein; M, Membrane protein; E, Envelop protein.
